# A Simplified Epicardial Ablation Strategy for Ventricular Tachycardia Integrating CT‐Derived Epicardial Adipose Tissue Into Three‐Dimensional Electroanatomic Mapping

**DOI:** 10.1111/jce.70382

**Published:** 2026-05-27

**Authors:** Koumei Onuki, Kenichi Hiroshima, Akihiro Isotani, Masato Fukunaga, Michio Nagashima, Kenji Ando

**Affiliations:** ^1^ Department of Cardiology Kokura Memorial Hospital Kitakyushu Japan

**Keywords:** epicardial ablation, epicardial adipose tissue, three‐dimensional electroanatomic mapping system, ventricular tachycardia

## Abstract

**Introduction:**

Epicardial adipose tissue (EAT) can attenuate electrograms and limit lesion formation during epicardial ventricular tachycardia (VT) ablation.

**Case Presentation:**

We developed a simplified workflow using predefined CT attenuation values to integrate EAT into the CARTO 3 system. We applied this approach in three patients with dilated cardiomyopathy undergoing epicardial VT ablation, where EAT visualization guided target selection, avoided ineffective ablation, and informed alternative endocardial strategies.

**Discussion:**

EAT‐integrated mapping improved the interpretation of epicardial substrates and influenced procedural decision‐making. This workflow may facilitate efficient assessment of epicardial ablation feasibility in complex VT cases.

## 1 Introduction

Epicardial catheter ablation is an important treatment option for ventricular tachycardia (VT), particularly in patients with nonischemic cardiomyopathy or prior failed endocardial ablation. However, its efficacy is frequently limited by epicardial adipose tissue (EAT), which attenuates electrogram amplitude and acts as a thermal barrier to radiofrequency energy delivery. Previous studies have shown that radiofrequency lesions fail to penetrate epicardial fat layers ≥ 3–3.5 mm, even with irrigated‐tip catheters [1], and that increasing EAT thickness progressively reduces lesion formation [2]. Computed tomography (CT)‐derived reconstruction of EAT and its integration into three‐dimensional (3D) electroanatomic mapping systems has been reported as a means to guide epicardial VT ablation [3]. However, conventional techniques often rely on labor‐intensive manual segmentation, limiting their routine clinical use. We therefore developed a simplified and reproducible workflow for delineating EAT using predefined CT attenuation values and integrating this EAT data set into a 3D electroanatomic mapping system. In this case series, we describe its application during epicardial VT ablation in three patients and demonstrate how EAT visualization influenced mapping interpretation and ablation strategy.

## 2 Case Report

### 2.1 Workflow for CT‐Derived EAT Reconstruction

The CARTO 3 System (Johnson & Johnson MedTech, Irvine, CA, USA) displays imported CT data by dividing the CT attenuation range between the maximum and minimum values into 4095 discrete increments. When CT images are exported with a CT value range of −2047 to +2047, a displayed value of approximately 2047 corresponds to a CT attenuation near 0 Hounsfield units (HU). Given that adipose tissue typically demonstrates attenuation values between approximately −200 and −30 HU, we determined that setting the display range on CARTO between 1847 and 2017 allowed selective visualization of adipose tissue. On the CT workstation (Ziostation2, Ziosoft Inc., Tokyo, Japan), a data set with CT attenuation values ranging from −2047 to 2047 was generated from coronary CT angiography acquired using Revolution CT (GE Healthcare, Waukesha, WI, USA) by applying the fitting and invert functions. The data set was then imported into the CARTO 3 System and displayed simultaneously with the electroanatomic map created during the ablation procedure. Spatial alignment was performed using anatomical landmarks such as the aorta, and EAT was overlaid onto voltage and activation maps for evaluation. Transparency and color settings were adjusted as needed for optimal visualization.

#### 2.1.1 Case 1

**Figure 1:**
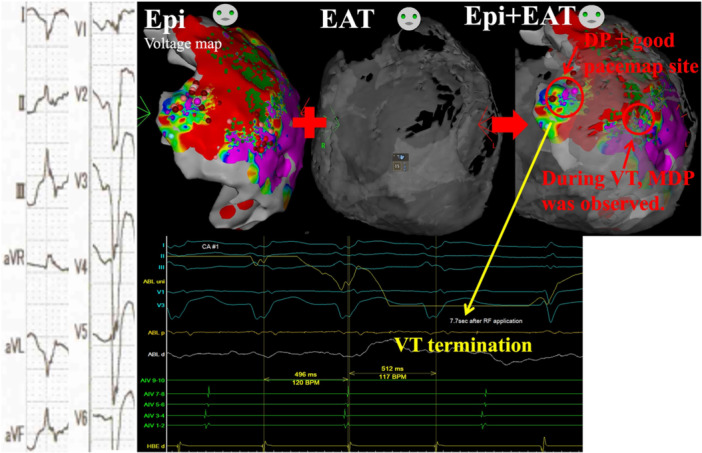
In Case 1, the 12‐lead electrocardiogram of the clinical ventricular tachycardia and voltage map with EAT visualization are shown. EAT is displayed in gray. The site where abnormal potentials are recorded was a region with relatively little EAT. The lower panel shows the intracardiac electrogram at the time of VT termination. DP, delayed potential; EAT, epicardial adipose tissue; Epi, epicardial; MDP, mid‐diastolic potential; VT, ventricular tachycardia.

A 68‐year‐old man with dilated cardiomyopathy underwent VT ablation for recurrent monomorphic VT. Endocardial voltage mapping revealed limited low‐voltage areas (LVA) without clear abnormal potentials. During epicardial mapping, an LVA with delayed potentials was identified at the LV basal anteroseptal region. During VT mapping, these delayed potentials became mid‐diastolic potentials (MDPs), and entrainment mapping demonstrated a post‐pacing interval nearly identical to the tachycardia cycle length. This region of interest was located at the border of the EAT, which was displayed as light purple areas in Figure 1. Pace mapping at this site produced a good pace map, and ablation at the same location terminated the clinical VT. The procedure was concluded after VT became non‐inducible.

#### 2.1.2 Case 2

**Figure 2:**
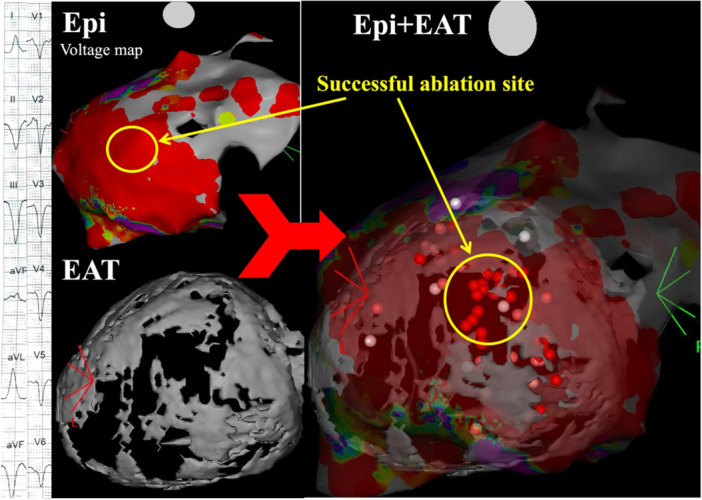
In Case 2, the 12‐lead electrocardiogram of the clinical ventricular tachycardia and 3D electroanatomic mapping with EAT visualization are shown. EAT is displayed in gray in the lower left panel. Abbreviations were similar to that of Figure 1.

A 67‐year‐old man with dilated cardiomyopathy had previously undergone epicardial VT ablation 11 years earlier and presented with recurrent VT. Endocardial substrate mapping demonstrated localized LVA but no delayed or late abnormal ventricular potentials. Epicardial mapping revealed a broad LVA in the LV posterolateral region, and there were no abnormal potentials. Integrated image analysis incorporating EAT clarified a region of interest within the LVA and demonstrated concordance with the site where Ripple bars accumulated. This region corresponded to the successful ablation target (yellow circle in Figure 2). VT became non‐inducible following ablation.

#### 2.1.3 Case 3

**Figure 3:**
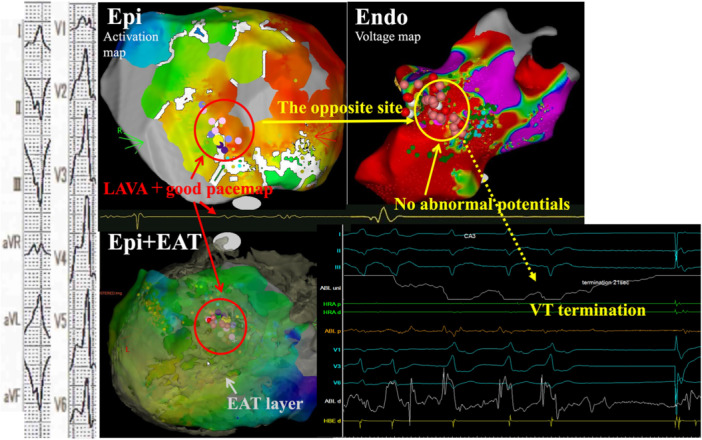
In Case 3, the 12‐lead electrocardiogram of the clinical ventricular tachycardia and voltage map, activation map, and activation map with EAT visualization are shown. EAT is displayed in gray in the lower panel. The center panel shows the intracardiac electrograms at the successful ablation site from both the epicardial and endocardial aspects. The lower right panel shows the intracardiac electrogram at the time of VT termination. Endo, endocardial; LAVA, late abnormal ventricular activation; other abbreviations were similar to that of Figures 1 and 2.

A 74‐year‐old man with dilated cardiomyopathy was referred after a failed VT ablation at another institution. Late abnormal ventricular activations (LAVAs) were identified on the epicardial map, and pace mapping at this site produced a waveform that matched the clinical VT. Merged CT imaging demonstrated that these abnormal epicardial potentials were located beneath the EAT layer. VT was not terminated by epicardial ablation at the site where these abnormal potentials were recorded. However, ablation delivered from the anatomically opposite LV endocardial side successfully terminated the clinical VT within 7.7 s. Given the presence of substantial EAT at the epicardial target sites, epicardial ablation was considered unlikely to achieve adequate lesion depth. This case highlighted the utility of EAT visualization in anticipating epicardial ablation failure and selecting an alternative therapeutic strategy (Figure 3).

## 3 Discussion

This case series illustrates a novel and simplified workflow for integrating CT‐derived EAT into 3D electroanatomic mapping during epicardial VT ablation. Prior studies have established that increasing EAT thickness is associated with reduced bipolar voltage amplitude and expansion of apparent LVA on epicardial maps [3]. Moreover, epicardial fat thickness exceeding 7 mm, as well as the presence of coronary arteries, has been identified as a major determinant of epicardial ablation failure.

Conventional CT‐derived EAT reconstruction techniques typically rely on manual segmentation of regions with attenuation values between −50 and −200 HU, followed by export and integration into mapping systems [3]. While effective, these methods are time‐consuming and may limit adoption in routine clinical practice. The workflow described here leverages intrinsic properties of the CARTO display algorithm and predefined CT attenuation ranges, allowing rapid creation and integration of EAT data sets without complex manual segmentation. Previous imaging studies have defined EAT using attenuation thresholds between −190 and −30 HU [4, 5]. In addition, the study analyzing the relationship between EAT and electrograms during AF defined fat as a CT attenuation range of −50 to −200 HU [6]. Our selected range (−200 to −30 HU) is consistent with these established definitions and was chosen to ensure comprehensive inclusion of adipose tissue while minimizing partial volume effects. This setting may improve imaging relevant to substrate interpretation, mapping, and ablation by enabling more selective visualization of EAT. However, no direct comparison with other attenuation ranges was performed in this study; therefore, the relative superiority of this threshold remains uncertain and warrants further investigation.

Importantly, visualization of EAT influenced procedural decision‐making in all three cases. In Case 1, the site where abnormal potentials were recorded corresponded to an area without EAT, which was useful for determining the ablation target site. In Case 2, EAT visualization may serve as a useful tool for narrowing down the region of interest within extensive epicardial LVA. Finally, Case 3 highlighted the utility of EAT visualization in anticipating epicardial ablation failure and selecting an alternative therapeutic strategy. These observations underscore that EAT‐integrated mapping may not only improve the interpretation of epicardial voltage maps but also help predict the feasibility and limitations of epicardial energy delivery.

## 4 Conclusion

We report a simplified and reproducible workflow for integrating CT‐derived EAT into 3D electroanatomic mapping during epicardial VT ablation. Visualization of EAT provided valuable insights into electrogram interpretation, anticipated limitations of epicardial energy delivery, and guided selection of ablation strategies. This approach may enhance procedural planning and decision‐making in complex VT ablation cases.

## Funding

The study was supported solely by departmental resources.

## Conflicts of Interest

The authors declare no conflicts of interest.

## Data Availability

The data that support the findings of this study are available from the corresponding author upon reasonable request.
